# ﻿A taxonomic study of four rare pteromalid genera: *Amblyharma* Huang & Tong, *Fusta* Xiao & Ye, *Nazgulia* Hedqvist and *Platecrizotes* Ferrière from the Eastern Palaearctic (Chalcidoidea, Pteromalidae, Pachyneurinae)

**DOI:** 10.3897/zookeys.1189.113982

**Published:** 2024-01-19

**Authors:** Ekaterina V. Tselikh, Jaehyeon Lee, Deok-Seo Ku

**Affiliations:** 1 Zoological Institute, Russian Academy of Sciences, St. Petersburg 199034, Russia Zoological Institute, Russian Academy of Sciences St. Petersburg Russia; 2 Department of Plant Medicine, Gyeongsang National University, Jinju 52828, Republic of Korea Gyeongsang National University Jinju Republic of Korea; 3 The Science Museum of Natural Enemies, Geochang 50147, Republic of Korea The Science Museum of Natural Enemies Geochang Republic of Korea

**Keywords:** Description, key, new record, new species, Pachyneurinae, parasitoid, redescription, taxonomy

## Abstract

The four morphologically similar genera *Amblyharma* Huang & Tong, 1993, *Fusta* Xiao & Ye, 2015, *Nazgulia* Hedqvist, 1973 and *Platecrizotes* Ferrière, 1934 from the Eastern Palaearctic are reviewed. Redescriptions of genera and all available types of Eastern Palaearctic species are provided. An identification key to genera is given. A new species from South Korea, *Platecrizotesjedii***sp. nov.** is described and illustrated.

## ﻿Introduction

Pteromalidae is one of the largest families of parasitic Hymenoptera, whose members are distributed in all zoogeographical regions of the world. It currently contains eight subfamilies and 415 genera (Burks et al. 2022). As a result of their large taxonomic and biological diversity, pteromalid wasps play a significant role as natural regulators of a number of phytophagous insects in natural and anthropogenic ecosystems and are potentially useful as biological control agents. Despite such high taxonomic diversity, abundance and significance, pteromalid wasps have not been sufficiently studied, in both taxonomic and faunistic investigations, and many species remain to be described.

This work is dedicated to four morphologically similar pteromalid genera that are poorly studied in the Eastern Palaearctic region: *Amblyharma* Huang & Tong, 1993, *Fusta* Xiao & Ye, 2015, *Nazgulia* Hedqvist, 1973 and *Platecrizotes* Ferrière, 1934, all belonging to the subfamily Pachyneurinae.

The monotypic genera *Amblyharma* Huang & Tong (type species *Amblyharmaanfracta* Huang & Tong, 1993) and *Fusta* Xiao & Ye (type species *Fustawuhuica* Xiao & Ye, 2015) are distributed only in the Eastern Palaearctic. The genus *Nazgulia* Hedqvist (type species *Nazguliapetiolata* Hedqvist, 1973) is recorded in both the Eastern and Western Palaearctic. *Platecrizotes* Ferrière (type species *Platecrizotessudanensis* Ferrière, 1934) contains four species distributed in the Palaearctic (*P.europaeus* Bouček, 1964 and *P.sudanensis* Ferrière, 1934), Oriental (*P.keralensis* Sureshan, Raseena Farsana & Nikhil, 2015), Afrotropical (*P.sudanensis* Ferrière) and Neotropical (*P.argentinensis* De Santis, 1988) regions (Noyes 2019).

Unfortunately, the biology of most species in these genera is unknown, but available records suggest that they are mostly primary parasitoids of dipterans in the families Drosophilidae – *Drosophila* sp. (*P.europaeus* and *P.keralensis*), Anthomyiidae – *Atherigonasoccata* Rondani, 1871 and Chloropidae – *Scoliophthalmusmicantipennis* Duda, 1935 (*P.sudanensis*), lepidopterans in the families Lasiocampidae – *Dendrolimus* sp. (*A.anfracta*) and Noctuidae – *Sesamiacretica* Lederer, 1857 (*P.sudanensis*), and coleopterans in the family Curculionidae – *Cryptobathyssetarius* Hustache, 1936 (*P.sudanensis*) (Noyes 2019).

The present paper is intended as a taxonomic study of the genera *Amblyharma* Huang & Tong, *Fusta* Xiao & Ye, *Nazgulia* Hedqvist and *Platecrizotes* Ferrière from the Eastern Palaearctic. These are small, rarely represented genera with only single specimens in collections. The original descriptions of the genera and species are incomplete and often contain significant errors; high-quality illustrations have not been published. The lack of contemporary keys for the identification of these pteromalids genera remains a major problem.

Therefore, the aim of this work is a comprehensive taxonomic study with redescriptions of genera and all available types of Eastern Palaearctic species and a description of a new species of *Platecrizotes* from South Korea. An identification key for these four genera is given.

## ﻿Materials and methods

The specimens examined in this study are deposited in the collections of the Institute of Zoology of the Chinese Academy of Sciences (Beijing, China; **IZAS**), the National Museum in Prague (Prague, Czech Republic; **NMPC**), the National Institute of Biological Resources (Incheon, Republic of Korea; **NIBR**), the Naturhistoriska Riksmuseet (Stockholm, Sweden; **NHRS**), and the Zoological Institute of the Russian Academy of Sciences (St Petersburg, Russia; **ZISP**).

Morphological terminology, including sculpture and wing venation, follows Bouček and Rasplus (1991), Gibson (1997), and Burks et al. (2022). The flagellum consists of two or three anelli, five or six funicular segments, and the four-segmented clava. The antennal formula includes the number of segments: scapus, pedicellus, anelli, funicular segments, claval segments. The following abbreviations are used: **POL** – posterior ocellar line, the minimum distance between the posterior ocelli; **OOL** – ocello-ocular line, the minimum distance between a posterior ocellus and compound eye; **C1–C4** – claval segments; **M** – marginal vein; **S** – stigmal vein; **PM** – postmarginal vein; **F1–F6** – funicular segments; **Mt2–Mt8** – metasomal tergites (Mt1 – petiole). The scape is measured without the radicle; the pedicel is measured in lateral view. The distance between the clypeal lower margin and the toruli is measured from the lower margins of the toruli. Eye height is measured as the maximum diameter, eye length as the minimum diameter. The mesosoma and metasoma are measured in lateral view, the latter including the ovipositor sheaths.

Specimens were examined using Olympus SZX12 and Nikon SMZ745T microscopes. Photographs were taken with a Canon EOS 70D digital camera mounted on an Olympus SZX10 microscope (ZISP specimens), and a Nikon DS-Ri1 digital camera mounted on a Nikon AZ100M microscope (IZAS specimens). The acquired images were then processed with Helicon Focus.

## ﻿Taxonomy

**Class Hexapoda Blainville, 1816**

**Order Hymenoptera Linnaeus, 1758**

**Family Pteromalidae Dalman, 1820**

### ﻿Subfamily Pachyneurinae Ashmead, 1904

The four genera *Amblyharma* Huang & Tong, *Fusta* Xiao & Ye, *Nazgulia* Hedqvist and *Platecrizotes* Ferrière are morphologically similar in having moderately depressed mesosoma with complete and shallow notauli (Figs 2, 7, 8, 10, 14, 16, 20), reticulate metapleuron (Figs 9, 14, 24), distinct petiole (Figs 1, 10, 16, 27), M of fore wing widened proximally (Figs 4, 12, 15, 19, 26). The differences between these genera are given in the key.

### ﻿Key to genera *Amblyharma* Huang & Tong, *Fusta* Xiao & Ye, *Nazgulia* Hedqvist and *Platecrizotes* Ferrière

**Table:** 

1	Antennal formula 11264 (Figs 6, 17)	**2**
–	Antennal formula 11354 (Figs 11, 25)	**3**
2	Pronotum with carina (Fig. 5). Lower margin of clypeus protruding (Fig. 3). F1 as long as F2 (Fig. 6). Propodeum with strong plicae (Fig. 1). Mesosoma (with propodeum) 1.50 times as long as wide (Fig. 2)	***Amblyharma* Huang & Tong, 1993**
–	Pronotum without carina (Fig. 16). Lower margin of clypeus not protruding (Fig. 17). F1 shorter than F2 (Fig. 17). Propodeum with weak plicae (Fig. 16). Mesosoma (with propodeum) 1.90 –2.00 times as long as wide (Fig. 16)	***Nazgulia* Hedqvist, 1973**
3	Clypeal margin emarginate (Fig. 11). Antennal toruli above ocular line (Fig. 11). Pronotum with carina. Right mandible with 3 teeth, left with 4 teeth. M of fore wing long and not strongly widened, 9.80 times as long as wide (Fig. 12). Hind tibia with one spur	***Fusta* Xiao & Ye, 2015**
–	Clypeal margin rounded (Fig. 22). Antennal toruli below ocular line (Fig. 22). Pronotum without carina (Fig. 21). Right and left mandibles with 4 teeth. M of fore wing short and strongly widened, less than 6.00 times as long as wide (Figs 19, 26). Hind tibia with two spurs	***Platecrizotes* Ferrière, 1934**

#### ﻿Genus Amblyharma

Taxon classificationAnimaliaHymenopteraPteromalidae

Huang & Tong, 1993

DFDB40F2-B3B8-5620-95EF-7902302FAC49

Amblyharma Huang &Tong, 1993: 395–397, 399–400. Type species Amblyharmaanfracta Huang & Tong, 1993, by original designation and monotypy.

##### Redescription.

Head without occipital carina. Gena without hollow at mouth corner; gena lamina absent. Lower margin of clypeus protruding and emarginate in the middle; tentorial pits indistinct (Fig. 3). Antennal formula 11264; anelli small, F1-F6 transverse, antennal clava not large, micropilosity area small and occupies the lower part of 2 last claval segments (Figs 3, 6). Antennal toruli situated above level of lower edges of eyes; antennal protuberance absent; scrobes shallow. Mandibles not visible.

**Figures 1–7.**
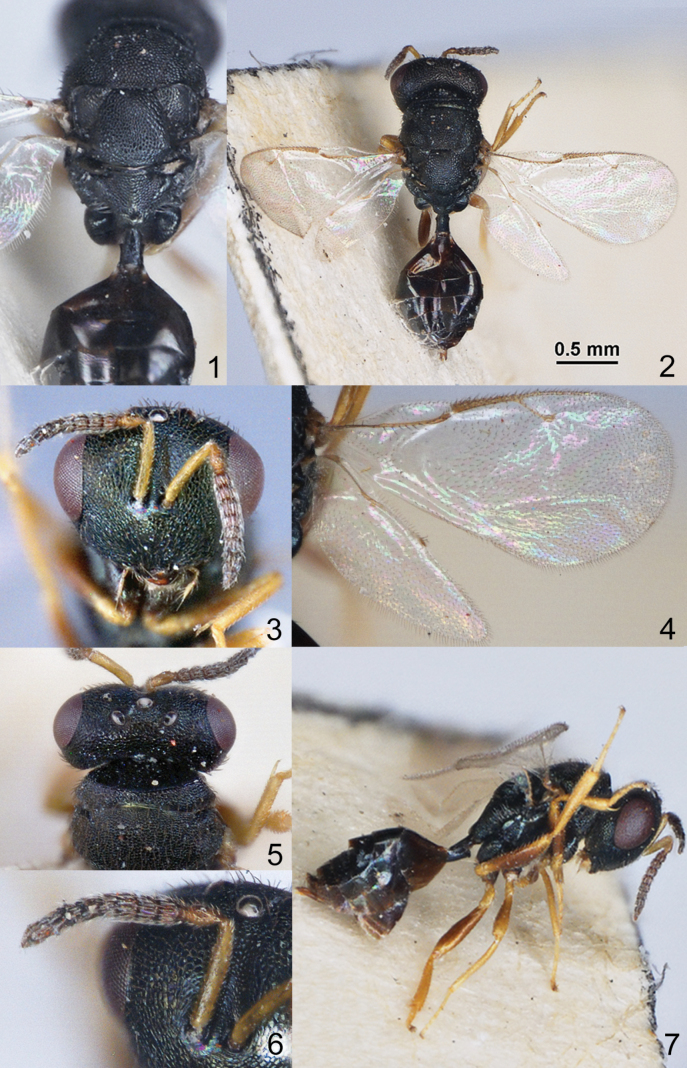
**1, 3***Amblyharmaanfracta* Huang & Tong, 1993, female, holotype **1** mesosoma and part metasoma, dorsal view **2** habitus, dorsal view **3** head, frontal view **4** wings **5** head, pronotum and mesoscutum, dorsal view **6** antenna **7** habitus, lateral view.

Mesosoma moderately depressed (Fig. 7). Pronotum little narrower than mesoscutum, with collar margin carinate. Notauli complete and shallow (Fig. 2). Scutellum moderately depressed, without conspicuous sublateral grooves, with distinct reticulate frenal area, but without frenal groove (Fig. 1). Metapleuron entirely reticulate. Propodeum with strong plicae; without costula and median carina, but middle part convex; nucha short and convex; propodeal spiracles near to front margin of sclerite (Fig. 1). Prepectus distinct, triangular, longer than tegula. Fore wing hyaline with distinct speculum; M widened proximally and tapering in distal part; M longer than S (Fig. 4). Hind coxa dorsally bare; hind tibia with one spur.

Metasoma on distinct reticulate, elongate petiole (Fig. 1). Metasoma ovate, flattened dorsally, shorter than combined length of mesosoma and head; Mt2 large with hind margin weak produced in middle (Figs 2, 7). Cerci with setae subequal in length. Hypopygium situated at one-third the length of metasoma. Ovipositor not much protruding.

##### Distribution.

Eastern Palaearctic.

#### ﻿ Amblyharma anfracta

Taxon classificationAnimaliaHymenopteraPteromalidae

Huang & Tong, 1993

9FDC6972-9CC5-54C8-A6E4-EBB2B8C650E3

Figs 1–7

Amblyharma anfracta Huang & Tong, 1993: 397. Holotype female (IZAS, examined).

##### Type material.

***Holotype***: female, “Hebei Province (Shijiazhuang), 1987.VIII.11”, “ex. *Carceliarasella* Baranoff (Li Wegao)”, “*Amblyharmaanfracta* ♀ Huang”, “HOLOTYPE”, “IOZ(E) 932939” (IZAS).

##### Description.

**Female.** Body length 2.30 mm; fore wing length 1.70 mm.

***Coloration*.** Head in dorsal view black, in frontal view dark green with metallic diffuse coppery lustre. Antenna with scape and pedicel yellowish-brown, flagellum brown. Mesosoma and all coxae black; propodeum dorsally dark green with metallic diffuse coppery lustre. All femora brown; tibiae and tarsi yellowish-brown. Fore wing hyaline, venation yellowish-brown. Metasoma dark brown; ovipositor sheaths yellowish-brown.

***Sculpture*.** Head reticulate; clypeus radially striate. Mesosoma and propodeum reticulate, nucha of propodeum alutaceous. Metasoma weakly alutaceous and shiny.

***Head*.** Head in dorsal view 2.20 times as broad as long and 1.15 times as broad as mesoscutum; in frontal view 1.28 times as broad as high. POL 1.25 times as long as OOL. Eye height 1.30 times eye length and 1.75 times as long as malar space. Distance between antennal toruli and lower margin of clypeus 0.95 times distance between antennal toruli and median ocellus. Antenna with scape 0.86 times as long as eye height and 1.13 times as long as eye length; pedicel 1.80 times as long as broad and 1.70 times as long as F1; combined length of pedicel and flagellum 0.74 times breadth of head; F1–F6 transverse with 1 row of sensilla; clava 2.10–2.20 times as long as broad, with small micropilosity area on C3 and C4.

***Mesosoma*.** Mesosoma 1.57 times as long as broad. Pronotum 0.40 times as long as mesoscutum. Scutellum 0.90 times as long as broad. Propodeum medially 0.68 times as long as scutellum; nucha 0.30 times length of propodeum. Fore wing 2.10–2.12 times as long as its maximum width; basal cell, cubital vein and basal vein pilose; speculum closed below; M 0.96–1.00 times as long as PM and 1.62–1.65 times as long as S.

***Metasoma*.** Metasoma 1.46 times as long as broad, 0.95 times as long as mesosoma and 0.74 times as long as mesosoma and head. Petiole 1.66 times as long as broad. Mt2 0.35 times median length of metasoma; Mt8 0.30 times longer than maximum width. Ovipositor sheath projecting slightly beyond apex of metasoma.

**Male.** The only one known male of this species is the one referred to in Huang & Tong, 1993. Unfortunately, it was not possible to study this specimen in the IZAS collection.

##### Distribution.

Peoples’ Republic of China (Hebei).

#### ﻿Genus Fusta

Taxon classificationAnimaliaHymenopteraPteromalidae

Xiao & Ye, 2015

535141E5-FA67-53E7-8142-D379EF245DD8

Fusta Xiao, Ye, 2015: 151–153. Type species Fustawuhuica Xiao & Ye, 2015, by original designation and monotypy.

##### Redescription.

Head without occipital carina. Gena without hollow at mouth corner; gena lamina absent. Lower margin of clypeus protruding and emarginate in the middle; tentorial pits indistinct (Fig. 11). Antennal formula 11354; anelli small, F1–F6 transverse, antennal clava not large, micropilosity area small and occupies the lower part of 2 last claval segments (Fig. 11). Antennal toruli situated above level of lower edges of eyes., Right mandible with 3 teeth, left with 4 teeth.

**Figures 8–13.**
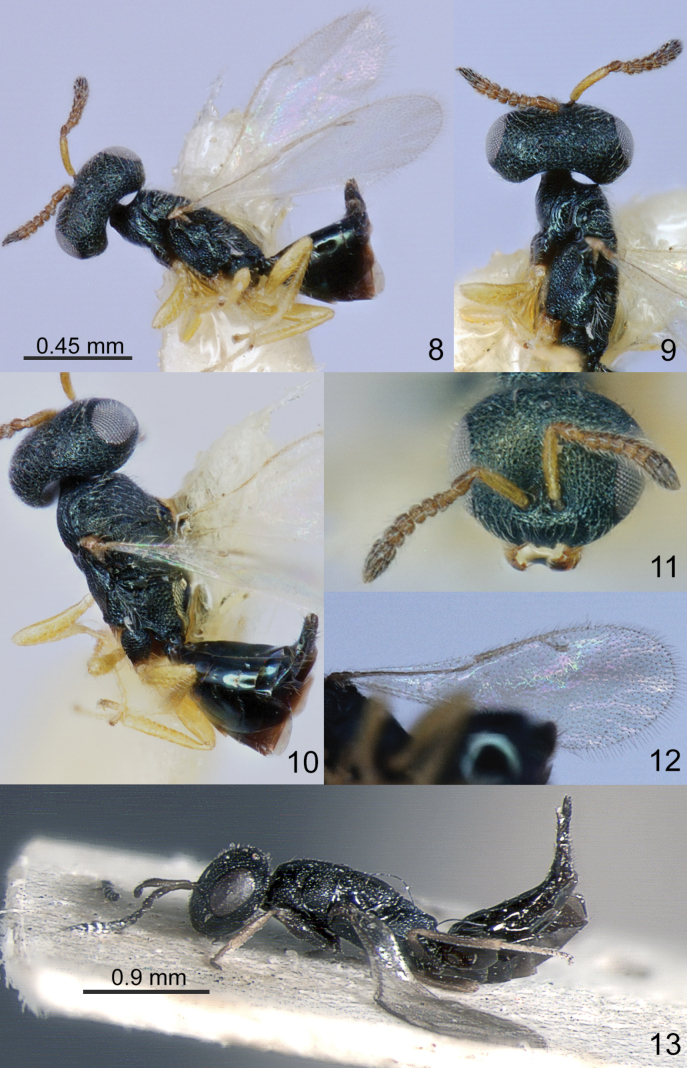
**8–12***Fustawuhuica* Xiao & Ye, 2015, female, holotype **8** head, dorsal view, mesosoma and metasoma, lateral view **9** head, dorsal view and mesosoma, lateral view **10** habitus, dorso-lateral view **11** head, frontal view **12** fore wing **13***Nazguliapetiolata* Hedqvist, 1973, female, holotype, habitus, lateral view.

Mesosoma moderately depressed (Fig. 8). Pronotum little narrower than mesoscutum, with collar margin carinate. Notauli complete and shallow (Fig. 10). Scutellum depressed, without conspicuous sublateral grooves, with distinct reticulate frenal area, but without frenal groove. Metapleuron entirely reticulate (Fig. 8). Propodeum without plicae, costula and median carina; nucha subglobose and reticulate; propodeal spiracles near to front margin of sclerite (Fig. 10). Prepectus distinct, triangular, shorter than tegula. Fore wing hyaline, without speculum; M widened proximally and tapering in distal part; M much longer than S (Fig. 12). Hind coxa dorsally bare; hind tibia with one spur.

Metasoma on distinct transverse petiole. Metasoma short ovate, flattened laterally, shorter than combined length of mesosoma and head (Fig. 8); Mt2 and Mt3 large, hind margin Mt2 arched in middle (Fig. 10). Cerci with setae subequal in length. Hypopygium situated at 0.6 length of metasoma. Ovipositor not much protruding.

##### Remarks.

The original description of the genus (Ye et al. 2015) indicated that both mandibles were with 3 teeth, but after studying the holotype it was concluded that the right mandible was with 3 teeth, the left with 4 teeth.

##### Distribution.

Eastern Palaearctic.

#### ﻿ Fusta wuhuica

Taxon classificationAnimaliaHymenopteraPteromalidae

Xiao & Ye, 2015

7AC1D530-0951-510C-9972-950CEDDE0110

Figs 8–12

Fusta wuhuica Xiao & Ye, 2015: 153–154. Holotype female (IZAS, examined).

##### Type material.

***Holotype***: female, “China: Anhui: Wuhu, viii.2011, rice fields”, “Coll. HU Hao-Yuan”, “*Fustawuhuica* Xiao et Ye, 2014”, “HOLOTYPE”, “IOZ(E) 1812583” (IZAS).

##### Description.

**Female.** Body length 1.30 mm; fore wing length 1.20 mm.

***Coloration*.** Head in frontal view dark green with metallic diffuse coppery lustre, in dorsal view dark blue-green with metallic diffuse coppery lustre; antenna with scape, pedicel, anelli and F1-F5 yellowish-brown, clava brown. Mesosoma, propodeum and all coxae dark blue-green with metallic diffuse coppery lustre; all femora, tibiae and tarsi yellow. Fore wing hyaline, venation yellowish-brown. Metasoma in dorsal view dark blue-green, in ventral view brown; ovipositor sheaths black.

***Sculpture*.** Head reticulate; clypeus radially striate. Mesosoma, propodeum with nucha reticulate; petiole weakly reticulate. Metasoma weakly alutaceous and shiny.

***Head*.** Head in dorsal view 1.90 times as broad as long and 1.33 times as broad as mesoscutum; in frontal view 1.25 times as broad as high. POL 0.92 times as long as OOL. Eye height 1.50 times eye length and 2.60 times as long as malar space. Distance between antennal toruli and lower margin of clypeus 0.60 times distance between antennal toruli and median ocellus. Antenna with scape 0.70 times as long as eye height and 1.07 times as long as eye length; pedicel 1.88 times as long as broad and 3.44 times as long as F1; combined length of pedicel and flagellum 0.78 times breadth of head; F1–F5 transverse with 1 row of sensilla; clava 2.00 times as long as broad, with small micropilosity area on C3 and C4.

***Mesosoma*.** Mesosoma 1.58 times as long as broad. Pronotum 0.80 times as long as mesoscutum. Scutellum 1.10 times as long as broad. Propodeum medially 0.90 times as long as scutellum; nucha 0.45 times length of propodeum. Fore wing 2.82 times as long as maximum width; basal cell, cubital vein, basal vein pilose; speculum absent; M 1.66 times as long as PM and 2.35 times as long as S.

***Metasoma.*** Metasoma 1.40 times as long as broad, 0.80 times as long as mesosoma and 0.60 times as long as mesosoma and head. Petiole 0.60 times as long as broad. Mt2 0.25 times median length of metasoma; Mt8 1.15 times longer than maximum width. Ovipositor sheath projecting slightly beyond apex of metasoma.

**Male.** Unknown.

##### Remarks.

The description of the species *F.wuhuica* by Xiao and Ye (2015) provides measurements that do not coincide with our measurements made during the study of the type material: body length 1.70 mm (redescription – 1.30 mm); head in dorsal view 3.17 times as broad as long (1.90); eye height 3.30 times eye length (1.50); fore wing 2.57 times as long as maximum width (2.82); M 1.33 times as long as P (1.66).

##### Distribution.

Peoples’ Republic of China (Anhui).

#### ﻿Genus Nazgulia

Taxon classificationAnimaliaHymenopteraPteromalidae

Hedqvist, 1973

7210DAA7-A3B4-5039-9ABE-614AB82A6BBF

Nazgulia Hedqvist, 1973: 239–240. Type species Nazguliapetiolata Hedqvist, 1973, by original designation and monotypy.

##### Redescription.

Head without occipital carina. Gena without hollow at mouth corner; gena lamina absent. Lower margin of clypeus not protruding, emarginate in the middle; tentorial pits indistinct (Fig. 17). Antennal formula 11264; anelli small, F1 transverse and shorter than F2, F2 longer than broad, F3-F6 subquadrate, antennal clava not large, micropilosity area small and occupies the lower part of 2 last claval segments (Fig. 17). Antennal toruli situated on level of lower edges of eyes; antennal protuberance absent; scrobes shallow. Both mandibles with 4 teeth (Fig. 17).

**Figures 14–19.**
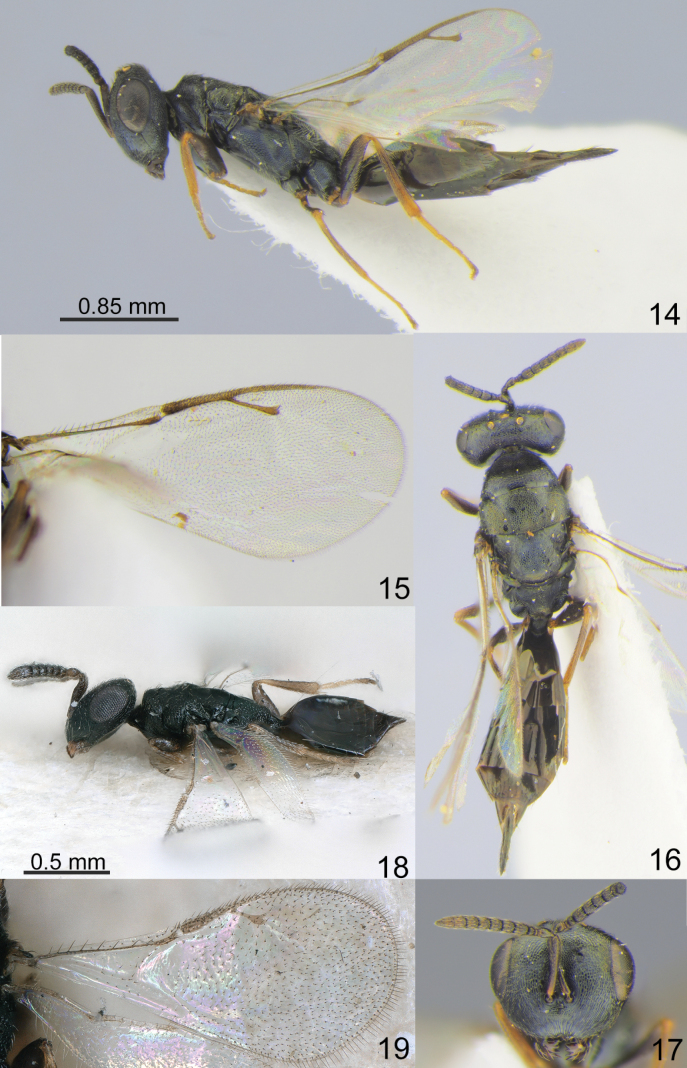
**14–17***Nazguliapetiolata* Hedqvist, 1973, female, non-type **14** habitus, lateral view **15** fore wing **16** habitus, dorsal view **17** head, frontal view **18, 19***Platecrizoteseuropaeus* Bouček, 1964, female, holotype **18** habitus, lateral view **19** fore wing.

Mesosoma moderately depressed (Figs 13, 14). Pronotum narrower than mesoscutum; collar margin not carinate. Notauli complete (Fig. 16). Scutellum depressed, without conspicuous sublateral grooves, with distinct reticulate frenal area and shallow frenal groove (Fig. 16). Metapleuron entirely reticulate (Fig. 14). Propodeum with weak plicae; costula and median carina absent; nucha subglobose and reticulate; propodeal spiracles near to front margin of sclerite (Fig. 16). Prepectus distinct triangular, longer than tegula. Fore wing hyaline, with speculum; M widened proximally and tapering in distal part; M slightly longer than S (Fig. 15). Hind coxa dorsally bare; hind tibia with one spur.

Metasoma on distinct petiole, longer than broad. Metasoma lanceolate, as long as combined length of mesosoma and head (Figs 13, 14); Mt2 large with hind margin arched in middle. Cerci with setae subequal in length. Hypopygium situated at one-half length of metasoma. Ovipositor not much protruding.

##### Remarks.

The original description of the genus by Hedqvist (1973) indicated that the notauli were incomplete, but after studying the holotype and additional non-type material it was concluded that the notauli are complete.

##### Distribution.

Palaearctic.

#### ﻿ Nazgulia petiolata

Taxon classificationAnimaliaHymenopteraPteromalidae

Hedqvist, 1973

34B3F77B-A9ED-56B2-8B8E-4B74404849EE

Figs 13 , 14–17

Nazgulia petiolata Hedqvist, 1973: 240. Holotype female (NMP, examined).

##### Type material.

***Holotype***: female, “Nrk. Asbro 25/5 1950 K: J. Hedqvist”, “HOLOTYPUS Nazgulia gen.n. petiolata sp.n. ♀ K-J Hedqvist det. 1973”, “NHRS-HEVA 000002235” (NHRS).

##### Additional material examined.

Russia: 1 female, “Sakhalin Prov., Sokol Vill., 7–9.VII.2011, E. Tselikh and D. Rachin” (ZISP).

##### Description.

**Female.** Body length 2.70–3.5 mm; fore wing length 1.90–2.10 mm.

***Coloration*.** Head, mesosoma and propodeum dark blue-green or black with metallic diffuse coppery lustre. Antenna with scape, pedicel and flagellum brown. All coxae dark blue-green or black with metallic diffuse coppery lustre; all femora dark brown with metallic blue-violet lustre; all tibiae and tarsi yellowish-brown. Fore wing hyaline, venation yellowish-brown. Metasoma dark brown partially with metallic blue-violet lustre; ovipositor sheaths black.

***Sculpture*.** Head reticulate; clypeus and malar space radially striate. Mesosoma, propodeum with nucha and petiole reticulate. Metasoma weakly alutaceous and shiny.

***Head*.** Head in dorsal view 2.17–2.19 times as broad as long and 1.19–1.20 times as broad as mesoscutum; in frontal view 1.20–1.22 times as broad as high. POL 1.00–1.09 times as long as OOL. Eye height 1.50 times eye length and 1.40–1.60 times as long as malar space. Distance between antennal toruli and lower margin of clypeus 0.60–0.64 times distance between antennal toruli and median ocellus. Antenna with scape 0.87–0.90 times as long as eye height and 1.30–1.40 times as long as eye length; pedicel 1.60–1.42 times as long as broad and 1.70–2.70 times as long as F1; combined length of pedicel and flagellum 0.85–0.90 times breadth of head; F1 transverse, F2 1.14–1.25 times as long as broad, F3-F6 subquadrate, all with 1 row of sensilla; clava 2.00–2.30 times as long as broad, with small micropilosity area on C3 and C4.

***Mesosoma*.** Mesosoma 1.84–1.89 times as long as broad. Pronotum 0.65–0.80 times as long as mesoscutum. Scutellum 0.85–0.90 times as long as broad. Propodeum medially 0.60–0.73 times as long as scutellum; nucha 0.40 times length of propodeum. Fore wing 2.32 times as long as its maximum width; basal cell partly or wholly pilose, cubital vein and basal vein pilose; speculum closed below; M 0.84–0.90 times as long as PM and 1.33–1.35 times as long as S.

***Metasoma*.** Metasoma 2.60–2.90 times as long as broad, 1.28–0.96 times as long as mesosoma and 0.76–1.00 times as long as mesosoma and head (metasoma is deformed in the specimens studied so the measurements are approximate). Petiole 1.75–2.00 times as long as broad. Mt2 0.20 times median length of metasoma; Mt8 1.15–1.20 times longer than maximum width. Ovipositor sheath projecting beyond apex of metasoma.

**Male.** Not studied.

##### Remarks.

One characteristic of this species is that the antenna has two anelli, but there is a tendency towards reduction in the size of F1 in some Palaearctic specimens (see Bouček and Rasplus 1991 and Fig. 17).

##### Distribution.

Netherlands, Sweden, Russia (Far East).

#### ﻿Genus Platecrizotes

Taxon classificationAnimaliaHymenopteraPteromalidae

Ferrière, 1934

A916571B-EDD1-56E0-B4CA-57BDB4FE9CC8

Platecrizotes Ferrière, 1934: 90. Type species Platecrizotessudanensis Ferrière, 1934, by original designation and monotypy.

##### Redescription.

Head without occipital carina. Gena without hollow at mouth corner; gena lamina absent. Lower margin of clypeus protruding and rounded; tentorial pits indistinct (Fig. 22). Antennal formula 11354; anelli small, F1–F5 transverse, antennal clava not large, micropilosity area small and occupies the lower part of 2 last claval segments. Antennal toruli situated above level of lower edges of eyes; antennal protuberance absent; scrobes shallow. (Fig. 25)Both mandibles with 4 teeth.

**Figures 20–27.**
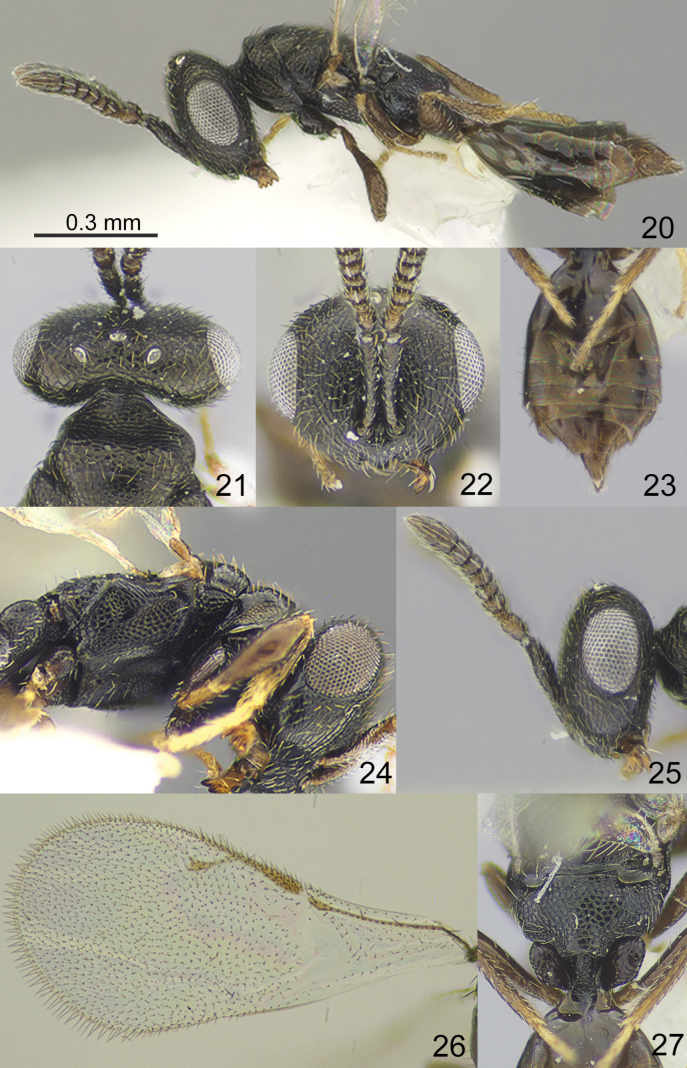
**20–27***Platecrizotesjedii* sp. nov., female, holotype **20** habitus, lateral view **21** head and pronotum, dorsal view **22** head, frontal view **23** metasoma, dorsal view **24** head and mesosoma, lateral view **25** head, lateral view and antenna **26** fore wing **27** propodeum and petiole, dorsal view.

Mesosoma depressed (Fig. 20). Pronotum narrower than mesoscutum; collar margin not carinate (Fig. 21). Notauli complete and shallow; metapleuron reticulate (Fig. 24). Scutellum depressed, without conspicuous sublateral grooves, frenal area and frenal groove. Metapleuron entirely reticulate. Propodeum with weak plicae indicated anteriorly; costula and median carina absent; nucha short and convex; propodeal spiracles near to front margin of sclerite (Fig. 27). Prepectus distinct, triangular, longer than tegula. Fore wing hyaline, with speculum; M widened proximally and tapering in distal part; M longer than S (Figs 19, 26). Hind coxa dorsally bare; hind tibia with two spurs.

Metasoma on distinct reticulate petiole, longer or shorter than broad (Fig. 27). Metasoma ovate, flattened dorsally, shorter than combined length of mesosoma and head; Mt2 and Mt3 large, hind margin Mt2 produced in middle (Figs 18, 20). Cerci with setae subequal in length. Hypopygium situated at one-quarter the length of metasoma. Ovipositor not much protruding.

##### Distribution.

Palaearctic, Oriental, Afrotropical and Neotropical regions.

#### ﻿ Platecrizotes jedii sp. nov.

Taxon classificationAnimaliaHymenopteraPteromalidae

57A7D2D1-7287-5F11-A898-B4F93A547FF7

https://zoobank.org/C8A41CAA-913C-41D0-AE62-A26A242B8F15

Figs 20–27

##### Type material.

***Holotype***: female, South Korea: “Gyeonggi-do, Pocheon-si, Soheul-eup, 37°45'29.2"N, 127°10'0.4"E, 15.VI.2015, Park, Choi, Nam, Shin, Kim” (NIBR). ***Paratype***: female, “Jeollabuk-do, Gunsan-si, Okdo-myeon, Sinsido-ri, malaise trap, 04–18.VIII.2017, H.G. Lee” (ZISP).

##### Description.

**Female.** Body length 1.10–1.30 mm; fore wing length 0.80–1.05 mm.

***Coloration*.** Head and mesosoma black. Antenna with scape black, pedicel and flagellum brown. All coxae black, all femora and tibiae brown, tarsi yellowish-brown. Fore wing hyaline, venation yellowish-brown. Metasoma dark brown partially with metallic coppery-violet lustre; ovipositor sheaths brown.

***Sculpture*.** Head reticulate; clypeus alutaceous. Mesosoma with pronotum and mesoscutum reticulate; axillae weakly reticulate; scutellum alutaceous or weakly alutaceous and shiny; propodeum reticulate, nucha alutaceous; petiole weakly reticulate. Metasoma weakly alutaceous and shiny.

***Head*.** Head in dorsal view 2.20–2.29 times as broad as long and 1.22–1.24 times as broad as mesoscutum; in frontal view 1.16–1.20 times as broad as high. POL 1.13–1.21 times as long as OOL. Eye height 1.52–1.54 times eye length and 1.80–2.00 times as long as malar space. Distance between antennal toruli and lower margin of clypeus 0.35–0.41 times distance between antennal toruli and median ocellus. Antenna with scape 1.00–1.05 times as long as eye height and 1.52–1.61 times as long as eye length; pedicel 1.14–1.21 times as long as broad and 1.30–1.40 times as long as F1; combined length of pedicel and flagellum 0.77–0.81 times breadth of head; F1-F5 transverse, all with 1 row of sensilla; clava 1.89–2.05 times as long as broad, with small micropilosity area on C3 and C4.

***Mesosoma*.** Mesosoma 1.76–1.80 times as long as broad. Pronotum 0.75–0.85 times as long as mesoscutum. Scutellum 0.85–0.90 times as long as broad. Propodeum medially as long as scutellum; nucha 0.20–0.25 times length of propodeum. Fore wing 2.20–2.23 times as long as maximum width; basal cell, cubital vein and basal vein pilose; speculum closed below; M 2.00–2.16 times as long as PM and 1.78–1.93 times as long as S.

***Metasoma*.** Metasoma 1.77–1.84 times as long as broad, 0.90–1.05 times as long as mesosoma and 0.78–0.86 times as long as mesosoma and head. Petiole 1.70–1.80 times as long as broad. Mt2 0.40–0.43 times median length of metasoma; Mt8 1.10–1.20 times longer than maximum width. Ovipositor sheath projecting slightly beyond apex of metasoma.

**Male.** Unknown.

##### Etymology.

The species is named in honour of the “Star Wars” character – “Jedi” of George Lucas.

##### Distribution.

Korean Peninsula.

##### Remarks.

This species is similar to *P.europaeus* Bouček, 1964 (Figs 18, 19) in having black coloration of the head and mesosoma; S of the fore wing with a relatively small stigma; lower margin of the clypeus strongly protruding. However, *Platecrizotesjedii* sp. nov. has the fore wing with PM shorter than S (vs PM longer than S), M 5.65–6.06 times as long as broad and the proximally widened part occupying 0.50 of the vein length (vs M 3.80–4.90 times as long as broad and proximally widened part occupying 0.80 of vein length), speculum closed below (vs open); petiole 1.70–1.90 times as long as broad (vs 0.50–0.60); and all tibiae brown (vs yellowish-brown).

## Supplementary Material

XML Treatment for Amblyharma

XML Treatment for Amblyharma anfracta

XML Treatment for Fusta

XML Treatment for Fusta wuhuica

XML Treatment for Nazgulia

XML Treatment for Nazgulia petiolata

XML Treatment for Platecrizotes

XML Treatment for Platecrizotes jedii

## ﻿Additional information

### Author ORCIDs

Ekaterina V. Tselikh https://orcid.org/0000-0002-9184-043X

Jaehyeon Lee https://orcid.org/0000-0002-3666-6029

Deok-Seo Ku https://orcid.org/0000-0002-6274-6479

### Data availability

All of the data that support the findings of this study are available in the main text.
